# Recent advances of NFATc1 in rheumatoid arthritis-related bone destruction: mechanisms and potential therapeutic targets

**DOI:** 10.1186/s10020-024-00788-w

**Published:** 2024-02-03

**Authors:** Hao Zheng, Yuexuan Liu, Yasi Deng, Yunzhe Li, Shiqi Liu, Yong Yang, Yun Qiu, Bin Li, Wenbing Sheng, Jinzhi Liu, Caiyun Peng, Wei Wang, Huanghe Yu

**Affiliations:** TCM and Ethnomedicine Innovation & Development International Laboratory, School of Pharmacy, Innovative Materia Medica Research Institute, Hunan University of Chinese Medicine, Changsha, 410208 China

**Keywords:** Rheumatoid arthritis, NFATc1, Bone destruction, RANKL/RANK, Osteoclast, Osteoclastogenesis

## Abstract

Rheumatoid arthritis (RA) is a chronic autoimmune inflammatory disease characterized by inflammation of the synovial tissue and joint bone destruction, often leading to significant disability. The main pathological manifestation of joint deformity in RA patients is bone destruction, which occurs due to the differentiation and proliferation of osteoclasts. The transcription factor nuclear factor-activated T cell 1 (NFATc1) plays a crucial role in this process. The regulation of NFATc1 in osteoclast differentiation is influenced by three main factors. Firstly, NFATc1 is activated through the upstream nuclear factor kappa-B ligand (RANKL)/RANK signaling pathway. Secondly, the Ca^2+^-related co-stimulatory signaling pathway amplifies NFATc1 activity. Finally, negative regulation of NFATc1 occurs through the action of cytokines such as B-cell Lymphoma 6 (Bcl-6), interferon regulatory factor 8 (IRF8), MAF basic leucine zipper transcription factor B (MafB), and LIM homeobox 2 (Lhx2). These three phases collectively govern NFATc1 transcription and subsequently affect the expression of downstream target genes including TRAF6 and NF-κB. Ultimately, this intricate regulatory network mediates osteoclast differentiation, fusion, and the degradation of both organic and inorganic components of the bone matrix. This review provides a comprehensive summary of recent advances in understanding the mechanism of NFATc1 in the context of RA-related bone destruction and discusses potential therapeutic agents that target NFATc1, with the aim of offering valuable insights for future research in the field of RA. To assess their potential as therapeutic agents for RA, we conducted a drug-like analysis of potential drugs with precise structures.

## Introduction

Rheumatoid arthritis (RA) is a chronic autoimmune disease that causes progressive joint bone destruction, potentially resulting in permanent disability. It is a leading cause of disability worldwide, with a prevalence rate of approximately 0.24–0.30%, being more common in women than in men (Finckh et al. 2022). Persistent synovial triggers the rapid division and proliferation of synovial cells in the early stages of RA, leading to synovial tissue hyperplasia. Inflammatory synovial cells and immune cells infiltrate the joints, subsequently resulting in the destruction of cartilage and bone (Firestein and McInnes 2017; Smolen et al. 2018).

**Fig. 1:**
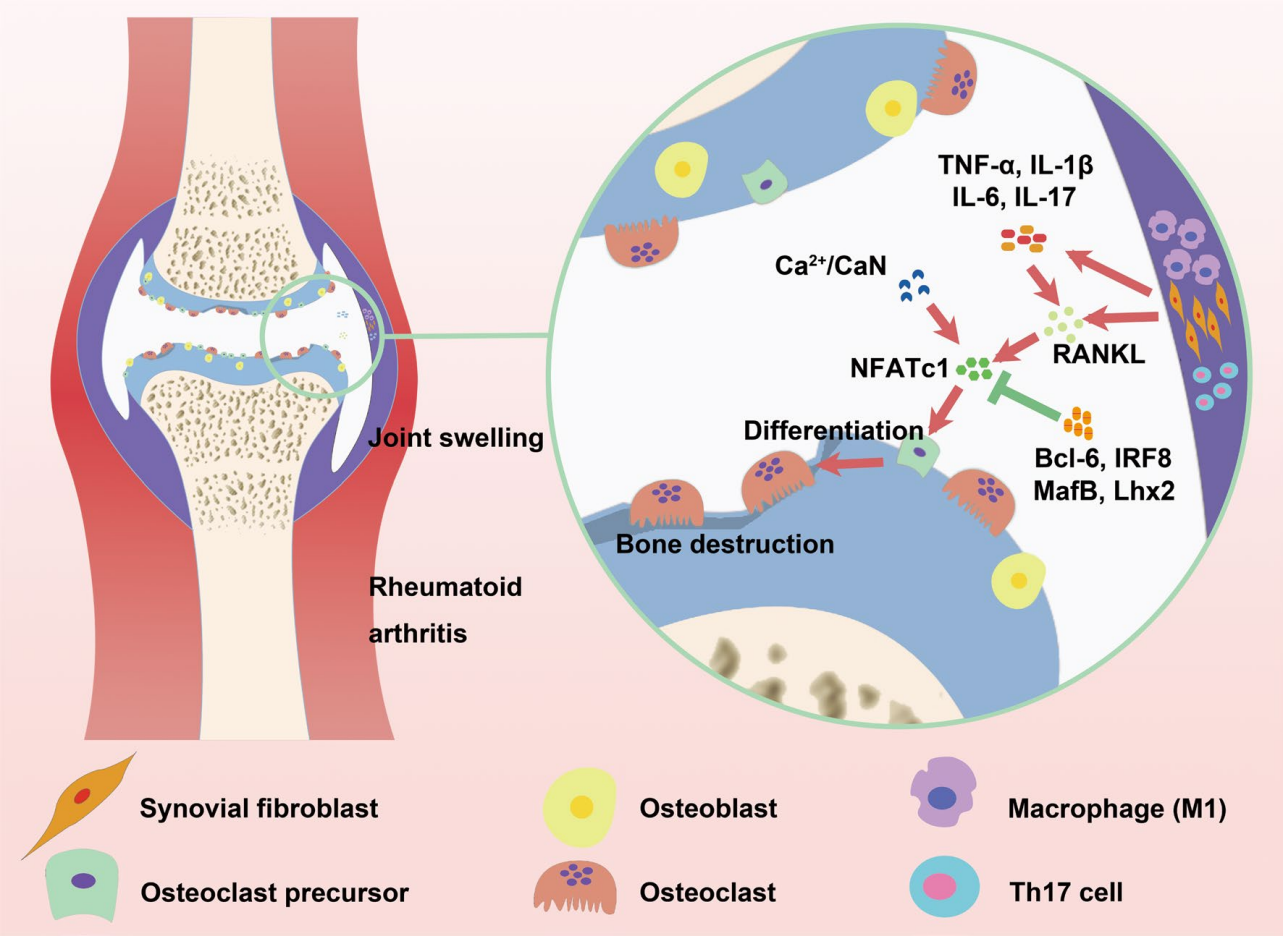
The development of RA bone destruction. In the process of RA, RAFLS, Th17, and M1 type macrophages secrete inflammatory factors such as TNF-α, IL-1β, IL-6, and IL-17, which promote RANKL secretion. Up-regulated RANKL stimulates the differentiation of osteoclast precursor cells into mature osteoclasts. The excessive proliferation of osteoclasts disrupts the delicate balance between osteoclasts and osteoblasts, leading to an imbalance in the bone microenvironment homeostasis. This dysregulation ultimately results in bone destruction characteristic of RA

Bone destruction is a significant consequence of the imbalance between osteoblasts and osteoclasts in joint bone tissue. Osteoclasts, which are multinucleated cells derived from the monocyte-macrophage lineage, play a crucial role in bone resorption and subsequent bone destruction (Jung et al. 2014). Studies have shown that bone destruction does not occur in mice without osteoclasts, such as tumor necrosis factor (TNF)-transgenic and serotransferrin-induced arthritic mice, suggesting that osteoclasts are the primary drivers of RA bone destruction (Pettit et al. 2001). The increase of osteoclasts is closely associated with the functions of many effector cells and immune cells in the local joints. These cells release a large amount of osteoclast differentiation factors under inflammatory stimulation, inducing the differentiation and maturation of osteoclasts. Among of them, RA fibroblast-like synoviocytes (RAFLS) secrete various pro-inflammatory cytokines, such as TNF-α, interleukin-1β (IL-1β), IL-6, and IL-17, chemokines, such as monocyte chemotactic protein-1 (MCP-1) and IL-8, and vascular endothelial growth factor (VEGF) to promote and maintain joint inflammation. Additionally, they secrete large quantities of the receptor activator of nuclear factor kappa-B ligand (RANKL) to stimulate the differentiation of osteoclast precursor cells into mature osteoclasts (Danks et al. 2016; Komatsu et al. 2021; McInnes and Schett 2011; Nygaard and Firestein 2020). Additionally, the infiltration of CD4^+^ T cells into the synovium is also an important pathological feature of RA, in which the balance of helper T cell 17 (Th17)/regulatory T cell (Tr cell) plays a pivotal role in osteoclast differentiation. Th17 cells enhance osteoclastogenic activity by producing IL-17 and induce osteoclast formation, mediating bone resorption by up-regulating RANKL (Chen et al. 2021). Furthermore, IL-17 increases the expression of pro-inflammatory cytokines, such as TNF-α and IL-1, and heightened the susceptibility of osteoclast precursor cells to RANKL (Funaki et al. 2018). Tr cells secrete cytokines, such as transforming growth factor-β (TGF-β), IL-10, and IL-4. Additionally, they employ cytotoxic T lymphocyte-associated antigen-4 (CTLA-4) to impede osteoclast differentiation (Yi et al. 2018) (Fig. 1). Macrophages, which are precursor cells of osteoclasts, can be classified into two subtypes: pro-inflammatory (M1) and anti-inflammatory (M2) (Zaidi and Cardozo 2018). In RA patients, macrophages primarily exhibit the M1 phenotype and secrete various pro-inflammatory cytokines, including TNF-α, IL-6, and IL-1β, which stimulate RANKL secretion. Conversely, M2 macrophages secrete cytokines such as IL-4 and IL-10, which impede osteoclast differentiation (Muñoz et al. 2020). Overall, in the pathogenesis of RA, these effector cells and immune cells regulate osteoclast generation and maturation by producing various cytokines, thereby promoting an increase in osteoclasts and disrupting the balance between osteoclasts and osteoblasts. Therefore, they are key factors leading to bone destruction.

Similarly, nuclear factor of activated T cells 1 (NFATc1) is a key transcription factor that induces osteoclast differentiation and maturation (Itzstein et al. 2011). Mice with NFATc1 gene deletion are unable to generate mature osteoclasts. Conversely, even in the absence of RANKL stimulation, ectopic expression of NFATc1 can effectively induce osteoclast differentiation, highlighting the indispensable role of NFATc1 in the process of osteoclast differentiation and maturation (Serfling et al. 2006; Takayanagi et al. 2002), NFATc1 can be activated by the RANKL/RANK signaling pathway, and this process is regulated by proteins downstream of the RANKL/RANK pathway, such as TNF receptor-associated factor 6 (TRAF6), NF-κB, and c-Fos. Imbalance in the transcription and negative regulation of NFATc1 after activation is a key factor in osteoclast maturation (Lorenzo 2017). Once NFATc1 is activated, calcium (Ca^2+^)-related co-stimulatory signaling pathways are activated to maintain the stability and amplification of NFATc1. Negative regulators of NFATc1, such as B-cell lymphoma 6 (Bcl-6), interferon regulatory factor 8 (IRF8), MAF bZIP transcription factor B (MafB), and LIM homeobox 2 (Lhx2), will be inhibited. Hence, NFATc1 plays a pivotal role in the development of bone destruction in RA. Suppression of NFATc1 results in a reduction of mature osteoclasts, which effectively mitigates bone damage and minimizes deformities in RA patients. Currently, there is a dearth of drugs specifically designed to target bone destruction in clinical settings. However, molecules that inhibit NFATc1 expression hold promise as potential therapeutic agents for addressing RA-induced bone destruction. Consequently, we have compiled an overview of the regulatory mechanisms involving NFATc1 in bone destruction and endeavored to identify potential bioactive compounds that could modulate NFATc1, thus providing valuable insights for the treatment of RA-related bone damage.

## The RANKL/RANK signaling pathway activates NFATc1

**Fig. 2:**
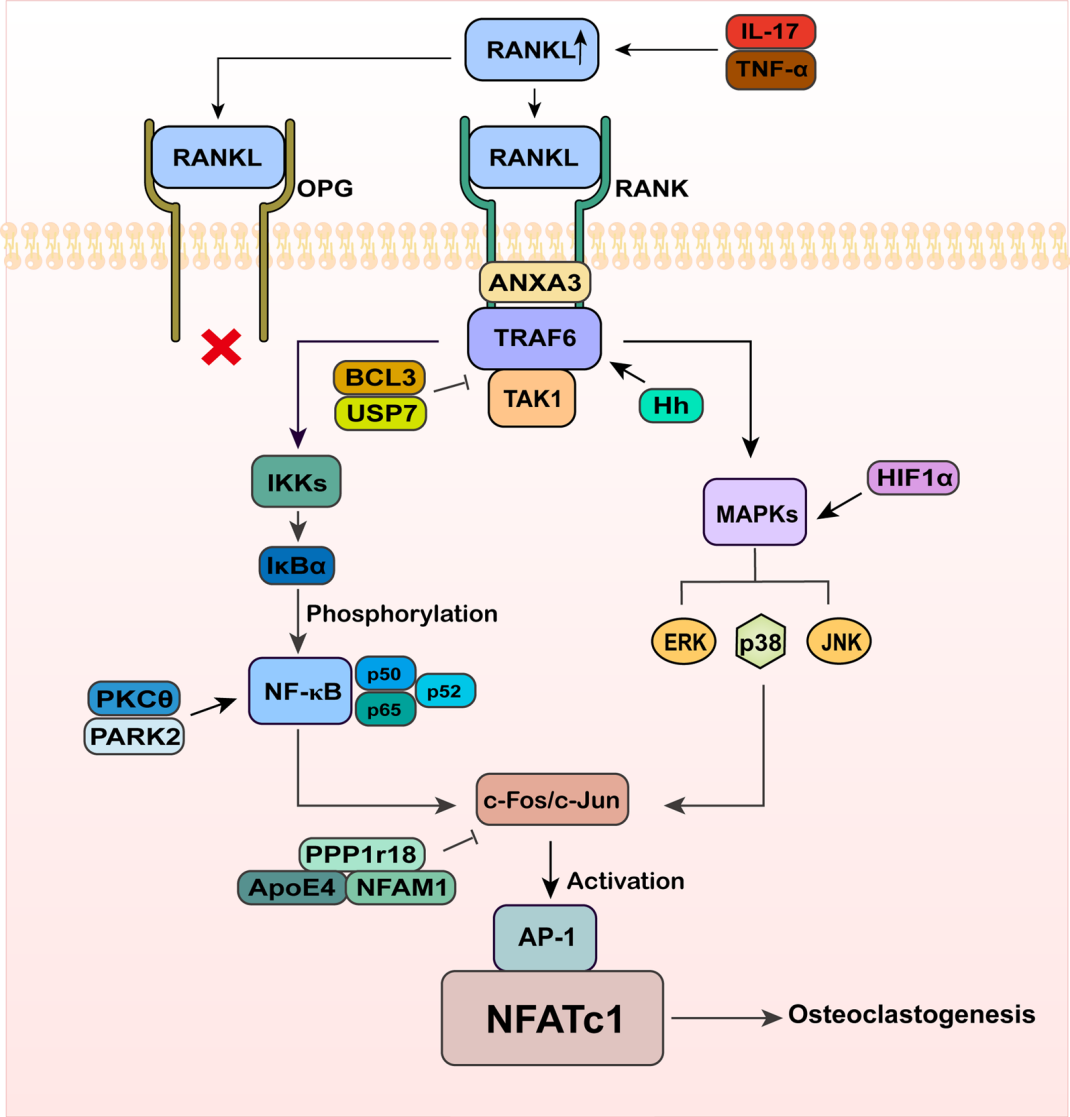
The RANKL/RANK signaling pathway activates NFATc1. RANKL binds to RANK, leading to the activation of downstream signaling pathways, including NF-κB and MAPK, through the involvement of TRAF6. The activated NF-κB and MAPK pathways mediate the expression of c-Fos and c-Jun, which in turn promote the activation of NFATc1. This activation of NFATc1 plays a crucial role in driving osteoclastogenesis, the formation and maturation of osteoclasts

The RANKL/RANK signaling pathway mediates the transcriptional activation of NFATc1, playing a crucial role in the regulation of osteoclast formation and differentiation (Amin et al. 2020). RANK, as the receptor for RANKL, is expressed on the surface of osteoclast precursor cells. Upon binding with RANKL, it triggers a series of downstream events, including the recruitment of adapter protein TRAF6, which in turn activates the NF-κB signaling pathway and mitogen-activated protein kinase (MAPK) pathways, such as the activation of Jun N-terminal kinase (JNK), extracellular regulated protein kinases (ERK), and p38 proteins (Omata and Tanaka 2011). The activation of the NF-κB pathway and MAPK pathway leads to upregulation of c-Fos and c-Jun expression. These proteins subsequently form a dimeric complex known as activator protein 1 (AP-1) (Ono and Nakashima 2018). AP-1 is recruited to the promoter region of the NFATc1 gene, where it activates the transcription of NFATc1 (Asagiri et al. 2005) (Fig. 2). Osteoprotegerin (OPG) is a decoy receptor for RANKL that competes with RANK for binding to RANKL. It inhibits osteoclast differentiation and function by interfering with the interaction between RANKL and RANK (Azizieh et al. 2019; Tsukasaki et al. 2020). During active RA, the expression of OPG decrease in the synovial tissue. Similarly, at sites of bone destruction where RANKL is abundantly expressed, the expression levels of OPG also decrease. As a result, the diminished levels of OPG lead to reduced competition with RANK, causing an elevated binding affinity between RANKL and RANK. Consequently, this promotes osteoclast differentiation and contributes to the progression of bone destruction in RA. Consequently, this promotes osteoclast differentiation and contributes to the progression of bone destruction in RA (Crotti et al. 2002; Pettit et al. 2006).

### RANKL/RANK

After binding with RANKL, RANK can not only signal osteoclast precursors through a cascade amplification reaction to activate NFATc1, promoting the differentiation and maturation of osteoclasts and resulting in bone destruction, but it can also trigger the activation of the RANKL/RANK pathway, activating NF-κB and stimulating effector cells such as RAFLS to secrete pro-inflammatory cytokines such as IL-6, IL-8, and PGE2, creating a sustained inflammatory environment that further promotes osteoclast differentiation and maturation, thereby exacerbating bone destruction (Amin et al. 2020). Studies have found that RANKL-deficient mice exhibit less severe bone destruction. On the other hand, increasing the expression of RANKL can promote the formation of osteoclasts in the joints and significantly exacerbate bone destruction (Pettit et al. 2001; Redlich et al. 2002). Silencing the RANKL gene using a lentiviral vector has been shown to substantially decrease NFATc1 levels in osteoclast precursor cells. Decreased NFATc1 expression directly reduces osteoclast production and inhibits osteolysis induced by wear particles (Zhang et al. 2017). G-protein coupled receptor 4 (LGR4) has been identified as a potential alternative receptor for RANKL. During the process of osteoclast differentiation, LGR4 competitively binds with RANKL through RANK, inhibiting RANKL/RANK signaling. This leads to downregulation of NFATc1 mRNA expression levels and its nuclear translocation, thereby suppressing osteoclast maturation (Luo et al. 2016; Renema et al. 2016; Yi 2016). It is worth noting that during the process of osteoclast differentiation, activation of the RANKL/RANK signaling pathway can directly induce the expression of LGR4. This may involve a self-regulatory mechanism in the bone microenvironment to prevent excessive transcription of NFATc1 and protect the joints (Jang et al. 2021; Okamoto et al. 2017). Wang et al. found that low levels of cadmium exposure may upregulate RANKL and TRAF6 expression to stimulate NFATc1 expression by inhibiting LGR4, consequently promoting osteoclast differentiation and formation (Wang et al. 2022b).

De Matos et al. conducted a study showing that Isoimperatorin (ISO), a bioactive furanocoumarin found in traditional Chinese herbal medicines, inhibited the combination of RANKL and RANK, resulting in decreased NFATc1 expression and suppressed osteoclast formation (Li et al. 2023). In another study by Fan et al., it was observed that ISO reduced the secretion of pro-inflammatory factors and inhibited the differentiation of human periodontal membrane fibroblasts (hPDLCs) into osteoclasts under RANKL stimulation (Fan et al. 2023). The mechanism of ISO’s action on human cells involves inhibiting the NF-κB and MAPK pathways, and its therapeutic concentration is five times higher than that for mouse cells. Xu et al. discovered that niloticin could directly bind to RANK with a low dissociation constant, blocking the interaction between RANKL and RANK. This led to the inhibition of NFATc1 expression and ultimately negatively regulated osteoclast formation (Xu et al. 2022). Additionally, (−)-epigallocatechin-3-gallate (EGCG), a biologically active polyphenol, was found to bind directly to RANKL, disrupted the interaction between RANKL and RANK, thus inhibiting NFATc1 expression and blocking osteoclastogenesis (Xu et al. 2021). Moreover, Xu found that Ellagic acid (EA), a naturally occurring polyphenolic compound, exhibited strong affinities for RANKL and RANK, interfering with their interaction. This resulted in the inhibition of NFATc1 expression, ultimately suppressing osteoclast differentiation and F-actin ring formation (Xu et al. 2020). Kostenuik et al. demonstrated that denosumab, a monoclonal antibody, could bind to RANKL and block the RANKL/RANK signaling pathway, leading to the inhibition of osteoclast formation (Kostenuik et al. 2009). Meng et al. discovered that IL-20 acted synergistically with RANKL to activate downstream signaling pathways, promoting NFATc1 expression and osteoclast formation. This effect was reversed by IL-20 inhibitors as it inhibited the interaction between RANKL and IL-20 (Meng et al. 2023). Furthermore, Tran et al. found that Rab11b reduced the surface abundance of RANK on osteoclast precursor cells and weakened NFATc1 activation, suggesting that Rab11b could serve as an inhibitor of osteoclast formation (Tran et al. 2020).

### TRAF6

TRAF6 is an intracellular adaptor protein. When RANKL binds to RANK, TRAF6 is recruited to RANK, forming a trimeric complex between RANK and TRAF6. This complex further activates the NF-κB and MAPK pathways through TGF-β-activated kinase 1 (TAK1), leading to signal amplification and activation of NFATc1. This process induces the formation of osteoclasts and promotes bone destruction. Therefore, TRAF6 plays a crucial role in osteoclast formation (Yu et al. 2021). The Hedgehog (Hh) signaling molecule promotes osteoclast differentiation by facilitating the cis-activation of TRAF6 and stabilizing TRAF6 protein. In vitro studies have shown that inhibitors of the Hh signaling pathway can counteract this effect and inhibit osteoclast formation. This suggests that targeting TRAF6 could be a potential therapeutic approach to regulate NFATc1 and treat bone destruction (Lu et al. 2023). Ubiquitin-specific peptidase 7 (USP7) is a deubiquitinase that can bind to TRAF6 and inhibit the TRAF6/TAK1 axis, leading to suppression of NFATc1 expression and consequently reducing osteoclastogenesis (Xie et al. 2023). Membrane-associated protein A3 (Annexin A3, ANXA3) is a member of the membrane-associated protein family involved in membrane trafficking and cancer development. It has been found to directly bind to both RANK and TRAF6. The formation of the RANK-ANXA3-TRAF6 complex accelerates osteoclast differentiation and maturation by promoting NFATc1 transcription and limiting its degradation. This suggests that the ANXA3/TRAF6 axis is an important target in the treatment of bone destruction (Lin et al. 2023b). Additionally, Deepak et al. found that the intraflagellar transport complex B protein (IFT80) promotes the degradation of TRAF6 by binding to the Casitas lineage B lymphoma proto-oncogene-B (Cbl-b), which in turn reduces osteoclastogenesis. Knockdown of IFT80 induces an increase in the level of TRAF6 by inducing an increase in the ubiquitylation of Cbl-b, leading to osteoclast information (Deepak et al. 2022). A20, also known as TNF-α-induced protein 3 (TNFAIP3), has been found to suppress TRAF6-dependent autophagy of osteoclast precursor cells, thus inhibiting osteoclast formation under hypoxic conditions (Yan et al. 2020). Liao et al. demonstrated that TNF-α-related apoptosis-inducing ligand (TRAIL) inhibits osteoclast activation by preventing RANKL-induced assembly of lipid rafts and recruitment of lipid rafts to TRAF6 (Liao et al. 2019). Wang et al. identified that B-cell chronic lymphatic leukemia protein 3 (BCL3) interacts with TRAF6 through its ankyrin-repeat domain, resulting in the inhibition of osteoclast precursor cell conversion to osteoclasts (Wang et al. 2018). Interestingly, Liu et al. reported contrasting results, showing that overexpression of TRAF6 enhances proliferation, migration, and osteogenesis of adipose-derived mesenchymal stem cells (ADSCs) via the Raf-Erk-Merk-Hif1a pathway. Indeed, the role of TRAF6 in osteoclast formation may not always be promoting. In cases of overexpression, TRAF6 can activate the Raf-Erk-Merk-Hif1a pathway, enhancing the osteogenic potential of ADSCs and promoting bone formation (Liu et al. 2023b).

Huang et al. demonstrated that TRAF-STOP, a small molecule inhibitor of TRAF6, effectively inhibits osteoclast formation by reducing TRAF6 expression, highlighting the potential of TRAF6 inhibitors in treating bone destruction (Huang et al. 2023). Ke et al. discovered that curcumin inhibits osteoclast differentiation and autophagy of osteoclast precursor cells, exerting an anti-bone destructive effect. The overexpression of TRAF6 restores curcumin’s effect, indicating the TRAF6-dependent nature of curcumin’s therapeutic action on bone destruction (Ke et al. 2023). Moreover, curcumin at a concentration 10 times higher than the therapeutic concentration in mice has been shown to inhibit the differentiation of preosteoclasts (derived from healthy human’s peripheral blood) to osteoclasts (von Metzler et al. 2009). Nie et al. found that exposure to fluoride (F) increased TRAF6 expression in rat bone tissue, while exposure to arsenic (As) decreased it, suggesting the potential of As in the treatment of bone destruction (Nie et al. 2023). Anzai et al. designed a tetravalent peptide called RANK-tet, which contains binding motifs of RANK and the C-terminal domain of TRAF6 (TRAF-C). RANK-tet specifically targets the RANK binding region of TRAF-C, effectively inhibiting osteoclast differentiation and suggesting its potential as a novel anti-bone destruction agent (Anzai et al. 2022). Liu et al. discovered that Tereticornate A (TA), a natural terpene ester compound, effectively inhibits osteoclast formation by suppressing TRAF6 and NFATc1 expression (Liu et al. 2022a). Chen et al. found that Mogrol, an aglycon of mogroside, inhibits osteoclast formation by blocking TRAF6 activation and reducing NFATc1 expression (Chen et al. 2022b). Wang et al. demonstrated that curcumenol (CUL), an antioxidant sesquiterpene isolated from *Curcuma zedoaria*, suppresses osteoclast formation by blocking the binding of inositol polyphosphate multikinase (IPMK) to TRAF6 (Wang et al. 2021). Zhi et al. reported that l-tetrahydropalmatine (l-THP) disrupts the interaction between RANK and TRAF6, inhibiting NFATc1 expression and suppressing osteoclast differentiation (Zhi et al. 2020).

### NF-κB

The NF-κB signaling pathway participate in osteoclast formation through TRAF6-dependent manner. TRAF6 specifically binds to the C-terminal cytoplasmic region of RANK, leading to the activation of NF-κB kinase inhibitors (IKKs) (Boyce and Xing 2007). IKKs (IKKα and IKKβ) phosphorylate specific sites on the NF-κB inhibitor IκB, resulting in the activation of NF-κB (Boyce and Xing 2007). Activated NF-κB is transported into the cell nucleus, where the p50 and p65 subunits induce increased expression of c-Fos and NFATc1. Subsequently, c-Fos interacts with NFATc1, promoting the transcription and expression of osteoclast genes, thereby facilitating osteoclast differentiation. In addition, simultaneous expression of NF-κB p50 and p52 is crucial for the differentiation of osteoclast precursor cells into osteoclasts. Mice with dual knockout of NF-κB p50 and p52 exhibit an increase in the number of osteoclast precursor cells but are unable to form osteoclasts, resulting in severe osteosclerosis. However, mice with single knockout of either NF-κB p50 or p52 are able to form osteoclasts (Franzoso et al. 1997; Iotsova et al. 1997). Aoki et al. cultured lymphoproliferative (aly/aly) mice with a functional deficiency mutation in the map3k14 gene, which is involved in the processing of p100 to p52 in the NF-κB pathway. Due to the reduced number of osteoclasts, these mice exhibited mild muscle atrophy, suggesting that targeting the NF-κB pathway to inhibit NFATc1 expression and osteoclast formation could serve as a therapeutic approach for treating bone resorption (Aoki et al. 2023). Sirtuin 2 (SIRT2), a nicotinamide adenine dinucleotide (NAD)-dependent protein deacetylase, has been implicated in osteoclast formation. Liver-specific deficiency of SIRT2 has been shown to inhibit osteoclast formation and mitigate bone loss in a mouse model of osteoporosis. Lin et al. demonstrated that SIRT2 deficiency in hepatocytes significantly reduced the nuclear translocation of NF-κB p65, suggesting that SIRT2 may promote osteoclast formation by regulating the nuclear translocation of NF-κB p65 (Lin et al. 2023a). Wang et al. found that Trimethylamine-N-oxide (TMAO) treatment increased oxygen species (ROS) production and significantly stimulated NF-κB p65 activation, resulting in the upregulation of c-Fos and NFATc1 genes and proteins, promoting osteoclast differentiation and inducing bone loss in mice (Wang et al. 2022c). PKCθ, a member of the PKC family, has been found by Wang et al. to induce monocyte-osteoclast differentiation and promote bone invasion through activation of the NF-κB/IL-1β pathway (Wang et al. 2023c). Cheng et al. discovered that Heat shock protein 90β (Hsp90β) binds to IKKβ, reducing its ubiquitination and proteasomal degradation, thereby activating NF-κB signaling and leading to bone destruction in mice (Cheng et al. 2023). Hong et al. found that overexpression of PARK2, a protein associated with Parkinson’s disease, promoted osteoclast formation through IKK and NF-κB activation (Hong et al. 2022).

Zhang et al. discovered that pirfenidone (PFD) effectively reduced RANKL-induced osteoclastogenesis by impairing NF-κB activation and suppressing NF-κB expression (Zhang et al. 2023b). Sheng et al. demonstrated that Safranal (Saf), a monoterpene aldehyde, attenuated osteoclast differentiation by inhibiting IκBα degradation through promoting NF-κB p65 deacetylation and inactivating IKK, thus interfering with NF-κB signaling (Sheng et al. 2023). Wu et al. identified that Strontium ranelate (SR) hindered osteoclast formation by inducing NF-κB pathway-dependent autophagy, thereby mitigating bone destruction in rats (Wu et al. 2023). Ding et al. revealed that EPZ015866, an inhibitor of protein arginine N-methyltransferase 5 (PRMT5), hindered osteoclast differentiation and bone resorption by impeding the nuclear translocation of NF-κB through blocking the dimethylation of NF-κB p65 subunit (Ding et al. 2023). Kuang et al. found that Surfactin (a biosurfactant derived from Bacillus subtilis) could inhibit osteoclast formation and promote osteogenic differentiation of bone marrow mesenchymal stem cells (BMSCs) by regulating NF-κB signaling pathway, effectively alleviating bone destruction in mice (Kuang et al. 2023). Zhan et al. revealed that isopsoralen suppressed RANKL-induced osteoclast formation by inhibiting the NF-κB signaling pathway (Zhan et al. 2023). Furthermore, Zhu et al. found that isopsoralen promoted the differentiation of human jawbone marrow mesenchymal cells into osteoblasts, suggesting its potential in restoring bone formation (Zhu et al. 2023). Zhuang et al. demonstrated that Avicularin (AL), a flavonoid and quercetin derivative, ameliorated osteoporosis by disrupting osteoclast formation through inhibiting the NF-κB pathway (Zhuang et al. 2023). Huang et al. discovered that dictamnine (DIC), a furoquinoline alkaloid, suppressed osteoclast formation, bone resorption, F-actin band formation, and osteoclast-specific gene expression by inhibiting the activity of nuclear factor erythroid2-related factor 2 (Nrf2), promoting the binding of Nrf2 and NF-κB, and blocking NFATc1 expression (Wong et al. 2022). Chen et al. found that Eltanexor (Elt), a selective nuclear-export inhibitor, prevented NF-κB activity by trapping IκBα in the nucleus and protecting it from proteasomal degradation, thereby impeding the translocation of IκBα and NF-κB p65. Consequently, the inhibition of NF-κB suppressed NFATc1 and c-Fos activity and resulted in the downregulation of genes and proteins associated with bone destruction (Chen et al. 2022a).

### MAPK

MAPK, also known as Mitogen-Activated Protein Kinase, refers to a group of intracellular protein kinases that primarily consist of three signaling pathways: ERK1/2, JNK, and p38 (Kim and Choi 2010). Among these pathways, ERK is vital for osteoclast differentiation, osteoclast differentiation and maturation induced by RANKL were significantly inhibited in cells where ERK2 was knocked down (Zhang et al. 2021a). While p38 and JNK become phosphorylated in response to RANKL stimulation and participate in the differentiation, growth, and function of osteoclasts. The phosphorylation levels of ERK, JNK, and p38 significantly increase during the differentiation of macrophages into osteoclasts under RANKL stimulation (Zhou et al. 2019). Similar to the TRAF6/NF-κB pathway, activation of TRAF6 leads to the activation of the apoptosis signal regulating kinase 1 (ASK1) kinase, which in turn promotes the phosphorylation of JNK, ERK, and p38, thereby activating the MAPK signaling pathway. The activated MAPK pathway facilitates NFATc1 transcription and induces osteoclast maturation through the phosphorylation of transcription factors such as c-Fos and c-Jun, as well as the regulation of AP-1 levels (Zhang et al. 2018). Wang et al. discovered that Hypoxia-inducible factor 1 alpha (HIF1α) significantly increased RANKL-mediated osteoclast differentiation in RAW264.7 cells by upregulating the MAPK pathway and then activating the expression of osteoclast-specific genes such as c-Fos and NFATc1 (Wang et al. 2022a). He et al. found that knockdown of the specific m6A-binding protein YT521-B homology domain family 1 (YTHDF1) reduced the phosphorylation levels of key proteins in the MAPK signaling pathways and destabilized RANKL mRNA by inhibiting the endoplasmic reticulum (ER) stress signaling pathway, thereby inhibiting osteoclast differentiation (He et al. 2022a).

Sun et al. found that discovered that Pteryxin (PTX), a natural coumarin found in the *Peucedanum* species, which belongs to the Apiaceae family, effectively blocked the MAPK and Ca^2+^-calcineurin-NFATc1 signaling pathways in osteoclasts. It inhibited NFATc1 expression and the expression of osteoclast-specific genes by reducing reactive oxygen species (ROS) levels in osteoclasts (Sun et al. 2023). Trang et al. demonstrated that citropten pretreatment inhibited RANKL-induced MAPK and PLCγ/Ca^2+^ signaling pathways, thereby inhibiting osteoclast differentiation in RAW264.7 cells (Trang et al. 2023). Jin et al. found that Oridonin (ORI), a tetracyclic diterpenoid compound isolated from *Rabdosia rubescens*, inhibited the MAPK/NF-κB pathway and induced intracellular ROS generation, thereby interfering with the differentiation of RAW264.7 cells into osteoclasts (Jin et al. 2023b). Ni et al. reported that Formononetin (FMN), a phytoestrogen belonging to the isoflavone family, suppressed the inflammatory response by inhibiting the phosphorylation of ERK and JNK (Ni et al. 2023). In addition, Yu et al. found that FMN inhibited osteoclast-specific gene expression and osteoclast formation by suppressing the MAPK signaling pathway (Yu et al. 2023b). Qin et al. demonstrated that Isosinensetin (Iss), a flavonoid mainly derived from citrus fruits, reduced intracellular ROS levels by activating Nrf2 and its associated antioxidant enzymes. It also inhibited the MAPK and NF-κB signaling pathways, thereby blocking osteoclast formation (Qin et al. 2023). Wang et al. discovered that BML-111, a synthetic lipoxin A4 agonist, effectively alleviated structural joint damage and inhibited osteoclast formation by reducing the activation of MAPK pathways (Wang et al. 2023b). Tan et al. found that toosendanin (TSN) targeted and interfered with the activation of the p38 subunit, thereby regulating the MAPK cascade and inhibiting osteoclast formation (Tan et al. 2023). Jiang et al. demonstrated that PD0325901, a specific inhibitor of ERK, inhibited the expression of c-Fos and NFATc1, and suppressed osteoclast differentiation in a time-dependent and dose-dependent manner (Jiang et al. 2023). Xing et al. found that astragalin (AST), a bioactive component of Rosa agrestis, negatively regulated the MAPK signaling pathway and inhibited the expression of c-Fos and NFATc1 at different stages, resulting in reduced bone destruction in mice (Xing et al. 2022). Moreover, Jia et al. found that AST could inhibit the expression of proteins related to bone destruction such as MMP-1, MMP-3, and MMP-13 in fibroblast-like synoviocytes derived from RA patients (MH7A cells) (Jia et al. 2019). Jin et al. found that GSK 650394 could block osteoclast differentiation by inhibiting the activation of MAPK signaling pathway, regulating intracellular redox status, and downregulating NFATc1 expression (Jin et al. 2022). Chen et al. reported that metformin hydrochloride (Met) inhibited osteoclast differentiation and reduced bone resorption by suppressing ERK phosphorylation (Chen et al. 2022c). Wang et al. found that Thiaplakortone B (TPB, a natural compound derived from the Great Barrier Reef sponge Plakortis lita) blocked multiple upstream pathways of osteoclast differentiation, including MAPK and NF-κB signaling pathways, which in turn inhibited NFATc1 expression and osteoclast formation (Wang et al. 2022d). Salvadori et al. discovered that KYMASIN UP, a new dietary product, inhibited osteoclast formation by reducing p38 MAPK activation, resulting in the downregulation of bone fracture markers (Salvadori et al. 2022). Qiu et al. found that neratinib, a small molecule compound, inhibited the expression of osteoclast-specific genes by inhibiting the MAPK pathway, thereby suppressing osteoclast differentiation as well as cartilage degradation and osteoclast formation (Qiu et al. 2022). He et al. demonstrated that 12-Deoxyphorbol-13-Hexadecanoate (DHD), one of the main bioactive components of *Stellera chamaejasme* L. (Lang Du), inhibited osteoclast-specific gene expression and NFATc1 activation by suppressing RANKL-induced MAPK and Ca^2+^ signaling pathways (He et al. 2022b). Long et al. found that epoxymicheliolide (EMCL), a derivative of parthenolide, reduced the transcription and expression of NFATc1 by inhibiting the phosphorylation of ERK1/2, thereby inhibiting osteoclast formation and bone resorption (Long et al. 2022).

### C-Fos/c-Jun

The interplay between c-Jun/c-Fos and the NFATc1 family is recognized as a crucial mechanism in osteoclast differentiation. Following phosphorylation, the nuclear protein C-Fos combines with c-Jun to form a heterodimer, resulting in the assembly of the AP-1 complex. This complex specifically binds to designated sites on the DNA promoter and enhancer regions of the NFATc1 gene. Through this interaction, extracellular signals are translated into the activation of the NFATc1 gene, facilitating the progression of osteoclast differentiation and maturation (Zhang et al. 1998). The primary role of c-Fos in osteoclasts is to promote NFATc1 expression and synergistically initiate transcriptional cascades. This leads to the activation of multiple target genes involved in osteoclast differentiation and maturation, ultimately influencing the maturation of precursor cells (Matsuo et al. 2004). Studies have shown that mice lacking c-Fos initially exhibit osteosclerosis due to the deficiency of osteoclast precursor cells, indicating the vital role played by c-Fos in osteoclast differentiation and formation (Matsuo and Ray 2004). During osteoclast differentiation, activated c-Jun transferred from the cytoplasm to the nucleus, inducing the activation of AP-1 and osteoclast-specific genes such as matrix metalloproteinases and alkaline phosphatases. This stimulation promotes the maturation, differentiation, survival, fusion, and activation of osteoclast precursor cells (Nie et al. 2016). Ethiraj et al. demonstrated that knockdown of NFAT activating protein with immunoreceptor tyrosine-based activation motif 1 (NFAM1) could inhibit NFATc1 expression in osteoclasts by attenuating the activities of c-Fos, phosphorylated c-Jun, and c-Jun N-terminal kinase (Ethiraj et al. 2021). Yasuda et al. found that overexpression of protein phosphatase 1 regulatory subunit 18 (PPP1r18) could target and reduce the phosphorylation and nuclear localization of c-Fos, thereby suppressing the transcriptional activity of NFATc1 (Yasuda et al. 2021). Noguchi et al. discovered that Apolipoprotein E4 (ApoE4) protein downregulated c-Fos, leading to the inhibition of NFATc1 expression and osteoclast-specific gene expression (Noguchi et al. 2018).

Huang et al. discovered that zoledronic acid (ZOL) effectively reduced the expression of c-Jun, c-Fos, and NFATc1 by inhibiting the phosphorylation of c-Jun N-terminal kinase. This mechanism led to the downregulation of dendritic cell-specific transmembrane proteins and other osteoclast-specific markers (Huang et al. 2022). Zhang et al. found that treatment with 650-nm low-level laser irradiation (LLLI) significantly decreased the expression of c-Jun and c-Fos in rats (Zhang et al. 2020) (Table 1).

**Table 1:** Diverse agents that regulate RANKL/RANK signaling pathway in osteolclastogenesis

Agent	Mechanism	Cells	Animals	Administered dose		References
				In vitro	In vivo	
ISO	Inhibited the combination of RANKL/RANK	hPDLCs (human) BMMs (C57BL/6 mice)	C57BL/6 mice	10–50 μM 2–10 μM	10 mg/kg	Fan et al. (2023), Li et al. (2023)
Niloticin	Blocked the interaction between RANKL and RANK	RAW264.7 cell	N/A	2.5–7.5 μM	N/A	Xu et al. (2022)
EGCG	Disrupted the interaction between RANKL and RANK	RAW264.7 cell	N/A	10–50 μM	N/A	Xu et al. (2021)
EA	Interfered with the interaction between RANKL and RANK	RAW264.7 cell	N/A	1–8 μM	N/A	Xu et al. (2020)
Denosumab	Blocked the RANKL/RANK signaling pathway	BMMs (C3H/HeN mice)	BDF1 mice	50 pM	0.2, 1.0, 5.0 mg/kg	Kostenuik et al. (2009)
IL-20 inhibitors	Inhibited the interaction between RANKL and IL-20	BMMs (Sprague–Dawley rats)	Sprague–Dawley rats	0–20 ng/mL	N/A	Meng et al. (2023)
Rab11b	Eliminated the surface abundance of RANK on osteoclast precursor cells	RAW-D cells BMMs (C57BL/6 mice)	N/A	10 pM	N/A	Tran et al. (2020)
TRAF-STOP	Inhibited TRAF6 expression	BMMs (C57BL/6 mice)	C57BL/6 mice	1 μM	N/A	Huang et al. (2023)
Curcumin	Inhibited TRAF6 expression	preosteoclasts (human) BMMs (C57BL/6 mice)	Tg-hRANKL mice	1–10 μM 15 nM	200 mg/kg	Ke et al. (2023), von Metzler et al. (2009)
As	Decreased TRAF6 expression	RAW264.7 cell	Wistar rats	0.1–10 μM	2.5, 5, 10 mg/kg	Nie et al. (2023)
RANK-tet	Targeted the RANK binding region of TRAF-C	BMMs (C57BL/6 mice)	N/A	10 μg/mL	N/A	Anzai et al. (2022)
TA	Suppressed TRAF6 and NFATc1 expression	RAW264.7 cell	N/A	2.5–7.5 μM	N/A	Liu et al. (2022a)
Mogrol	Blocked TRAF6 activations and decreased NFATc1 expression	BMMs (C57BL/6 mice)	C57BL/6 mice	5–20 μM	10 mg/kg	Chen et al. (2022b)
CUL	Blocked the binding of IPMK to TRAF6	BMMs (C57BL/6 mice)	C57BL/6 mice	25–100 μM	2.5, 10 mg/kg	Wang et al. (2021)
l-THP	Blocked the interaction between RANK and TRAF6	RAW264.7 cell BMMs (C57BL/6 mice)	C57BL/6 mice	4.75–19.00 μg/mL	N/A	Zhi et al. (2020)
PFD	Decreased the effectiveness of NF-κB activation and inhibited NF-κB expression	BMMs (C57BL/6 mice)	C57BL/6 mice	200–800 μM	60, 120 mg/kg	Zhang et al. (2023b)
Saf	Interfered with NF-κB signaling	BMMs (C57BL/6 mice)	C57BL/6 mice	5–20 μM	10, 20 mg/kg	Sheng et al. (2023)
SR	Mediated NF-κB pathway-dependent autophagy	BMMs (Sprague–Dawley rats)	Sprague–Dawley rats	2 mM	900 mg/kg	Wu et al. (2023)
EPZ015866	Blocked the demethylation of NF-κB p65 subunit	RAW264.7 cell BMMs (C57BL/6 mice)	N/A	20–1000 nM	N/A	Ding et al. (2023)
Surfactin	Regulated NF-κB signaling pathway	BMMs (C57BL/6 mice)	C57BL/6 mice	10–1000 nM	4, 20 mg/kg	Kuang et al. (2023)
Isopsoralen	Suppressed NF-κB signaling pathway	Jawbone marrow mesenchymal cells (human) BMMs (C57BL/6 mice)	N/A	1 μM 10–30 μM	N/A	Zhan et al. (2023), Zhu et al. (2023)
AL	Inhibited NF-κB signaling pathway	RAW264.7 cell BMMs (C57BL/6 mice)	C57BL/6 mice	10–300 μM	1.25, 5 mg/kg	Zhuang et al. (2023)
DIC	Inhibited the activity of Nrf2 and NF-κB	RAW264.7 cell	C57BL/6 mice	50–150 μM	10, 20 mg/kg	Wong et al. (2022)
Elt	Blocked the translocation of IκBα and NF-κB p65	BMMs (C57BL/6 mice)	C57BL/6 mice	25–100 nM	0.075, 0.15 mg/kg	Chen et al. (2022a)
PTX	Blocked MAPK and Ca^2+^-calcineurin-NFATc1 signaling pathways	BMMs (C57BL/6 mice)	C57BL/6 mice	5–20 μM	5, 10 mg/kg	Sun et al. (2023)
Citropten	Inhibited MAPK and PLCγ/Ca^2+^ signaling pathways	RAW264.7 cell	N/A	5–40 μM	N/A	Trang et al. (2023)
ORI	Inhibited MAPK/NF-κB pathway and activated intracellular ROS generation	RAW264.7 cell BMMs (SD rats)	N/A	3.38 μM	N/A	Jin et al. (2023b)
FMN	Suppressed MAPK signaling pathway	BMMs (C57BL/6 mice)	C57BL/6 mice	5–40 μM	10, 20 mg/kg	Ni et al. (2023); Yu et al. (2023b)
Iss	Inhibited MAPK signaling pathway	BMMs (C57BL/6 mice)	C57BL/6 mice	1–10 μM	5, 10 mg/kg	Qin et al. (2023)
BML-111	Reduced the activation of MAPK pathways	BMMs (C57BL/6 mice)	C57BL/6 mice	25–100 μM	1 mg/kg	Wang et al. (2023b)
TSN	Interfered with p38 subunit activation and regulated the MAPK cascade	RAW264.7 cell BMMs (C57BL/6 mice)	C57BL/6 mice	2–8 nM	0.3, 0.6 mg/kg	Tan et al. (2023)
PD0325901	Inhibited ERK activation	BMMs (C57BL/6 mice)	C57BL/6 mice	0.32–1.28 nM	5, 10 mg/kg	Jiang et al. (2023)
AST	Negatively regulated MAPK signaling	MH7A cells (human) BMMs (C57BL/6 mice)	C57BL/6 mice	50–200 μM 50–100 μM	20 mg/kg	Jia et al. (2019), Xing et al. (2022)
GSK 650394	Inhibited the activation of MAPK signaling pathway	BMMs (C57BL/6 mice)	C57BL/6 mice	1–5 μM	10, 30 mg/kg	Jin et al. (2022)
Met	Suppressed ERK phosphorylation	BMMs (C57BL/6 mice)	N/A	200–400 μM	N/A	Chen et al. (2022c)
TPB	Blocked MAPK and NF-κB signaling pathways	BMMs (C57BL/6 mice)	C57BL/6 mice	1–10 μM	1 mg/kg	Wang et al. (2022d)
KYMASIN UP	Reduced p38 MAPK activation	RAW264.7 cell	N/A	12.5–100 μg/mL	N/A	Salvadori et al. (2022)
Neratinib	Inhibited the MAPK pathway	BMMs (C57BL/6 mice)	C57BL/6 mice	3.13–12.5 nM	5, 10 mg/kg	Qiu et al. (2022)
DHD	Suppressed MAPK and Ca^2+^signaling pathway	BMMs (C57BL/6 mice)	C57BL/6 mice	0.25–2 μM	2 mg/kg	He et al. (2022b)
EMCL	Inhibited the phosphorylation of ERK1/2	BMMs (C57BL/6 mice)	C57BL/6 mice	0.625–2.5 μM	2.5 mg/kg	Long et al. (2022)
ZOL	Reduced c-Jun and c-Fos expression	RAW264.7 cell	N/A	0.1–5 μM	N/A	Huang et al. (2022)
LLLI	Decreased c-Jun and c-Fos expression	N/A	Sprague–Dawley rats	N/A	N/A	Zhang et al. (2020)

In summary, the RANKL/RANK signaling pathway is the primary driver of NFATc1 activation. Targeting this pathway to inhibit NFATc1 activation remains a key focus in the study of bone destruction. Various agents that target the RANKL/RANK signaling pathway have shown great potential for RA treatment.

## The Ca^2+^-related co-stimulation signaling pathway mediates NFATc1 amplification

**Fig. 3:**
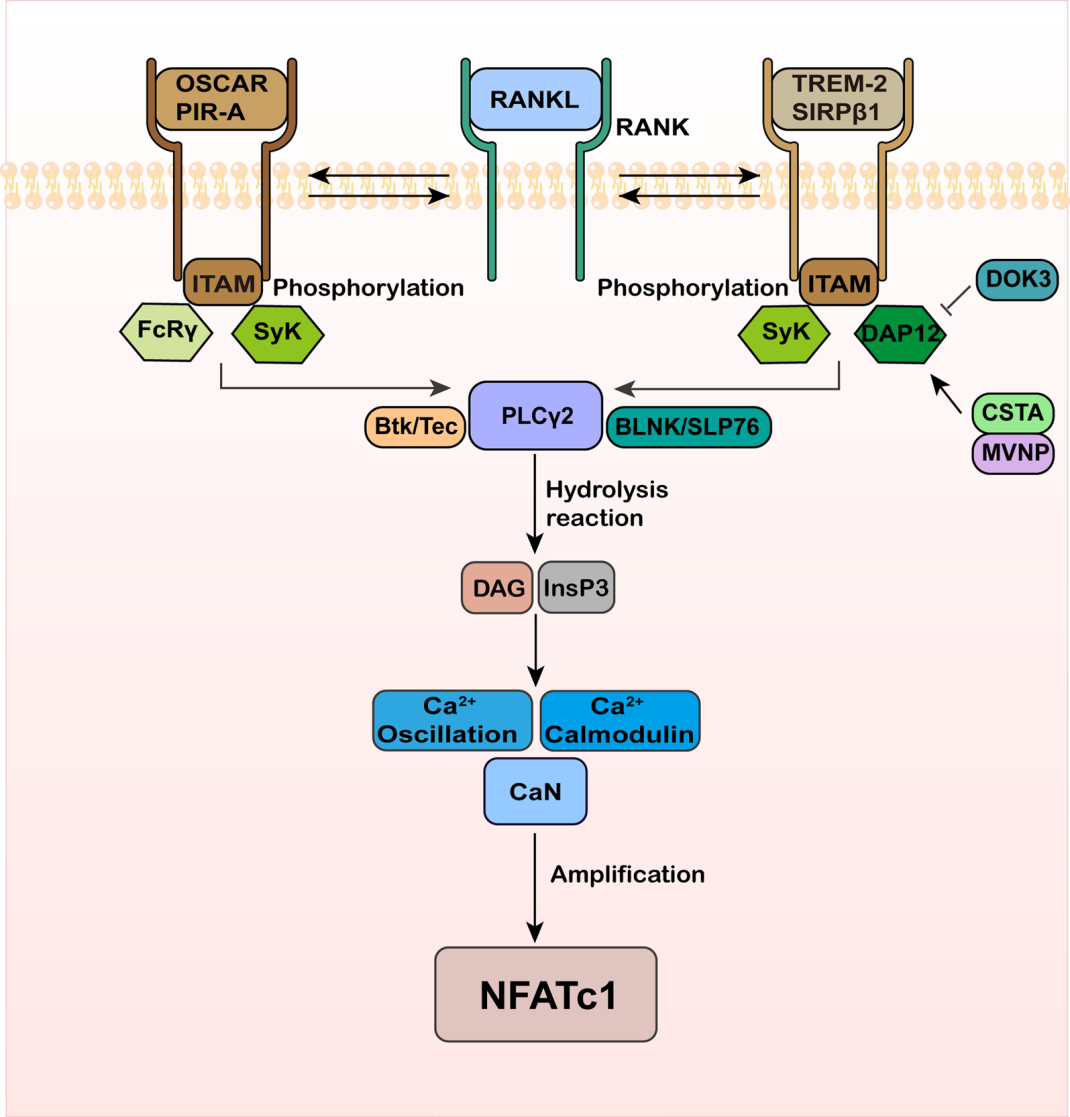
RANK collaborates with co-stimulatory receptors to facilitate the transmission of the Ca^2+^ signaling, sustaining the amplification of NFATc1. These co-stimulatory receptors include SIRPβ1, OSCAR, TREM-2, PIR-A, and FcRγ. When ITAM is phosphorylated, it binds to PLCγ2 and Syk, activating the immune receptors. Specifically, OSCAR and PIR-A bind to FcRγ, while TREM-2 and SIRPβ1 bind to DAP12. These interactions promote the transmission of the Ca^2+^ signaling and the subsequent amplification of NFATc1

Continuous transcription of NFATc1 is mainly maintained by the Ca^2+^ and CaN pathways. RANK, along with its co-stimulatory receptors on the cell membrane of osteoclast precursors, contributes to the stable amplification of NFATc1 by regulating the transduction of Ca^2+^ signaling. These co-stimulatory receptors include signal trigger receptors expressed on bone marrow cells 2 (TREM-2)/osteoclast-associated receptors (OSCAR), regulatory protein β-1 (SIRPβ1)/paired immunoglobulin receptors A (PIR-A), and the Fc receptor γ chain (FcRγ) (Asagiri and Takayanagi 2007; Okamoto et al. 2017; Tsukasaki and Takayanagi 2019). Downstream molecular signals such as the DNAX activating protein (DAP12) and FcRγ, which contain immunoreceptor tyrosine-based activation motifs (ITAMs), bind to these co-stimulatory receptors and activate Ca^2+^ signaling (Koga et al. 2004; Negishi-Koga et al. 2015). Phosphorylation of ITAMs triggers the recruitment of splenic tyrosine kinase (Syk), which subsequently activates adaptor proteins like B cell linker protein (BLNK) and SH2-containing leukocyte protein (SLP76). BLNK/SLP76, in turn, recruits Tec kinase Btk/Tec (phosphorylated by RANK) and phospholipase Cγ (PLCγ) to form a scaffold for osteoclast signaling complexes. This complex scaffold is crucial for the effective activation of Ca^2+^ signaling, ultimately promoting the transcription of NFATc1 and maintaining its amplification (Shinohara et al. 2008) (Fig. 3).

### Co-stimulatory receptors of RANK

In osteoclast precursor cells, ITAM phosphorylation stimulates the binding of PLCγ2 and Syk, which in turn activate the immunoreceptors OSCAR/PIR-A and TREM-2/SIRPβ-1. Upon activation, OSCAR/PIR-A binds to FcRγ, and TREM-2/SIRPβ-1 binds to DAP12, which is involved in the activation of Ca^2+^ signaling (Koga et al. 2004). It was discovered that mice deficient in DAP12 had increased osteogenesis. Additionally, mice deficient in both FcRγ and DAP12 experienced significant bone sclerosis due to decreased osteoclast differentiation (Kamimura et al. 2015). These findings suggest that FcRγ and DAP12 can promote osteoclast differentiation by participating in the activation of the Ca^2+^ signaling and inducing NFATc1 amplification. In the absence of RANKL stimulation, individual ITAM stimulation is not sufficient to activate the Ca^2+^ signaling. However, under the stimulation of RANKL, immune receptors OSCAR/PIR-A and TREM-2/SIRPβ-1 form receptor complexes with RANK and synergistically activate Ca^2+^ signaling, thereby promoting osteoclast differentiation by inducing NFATc1 amplification. Koga et al. established a co-stimulation model during osteoclast differentiation, confirming that the combined action of ITAM signaling and RANK signaling is a necessary condition for inducing NFATc1, which is the key transcription factor involved in osteoclast formation (Koga et al. 2004; Negishi-Koga and Takayanagi 2009).

Silencing cystatin A (CSTA) reduces the expression of DAP12 and TREM-2, thereby inhibiting osteoclast differentiation. Conversely, overexpression of CSTA can reverse this phenomenon and promote osteoclast differentiation (Wei et al. 2022). Cai et al. discovered that downstream of kinase-3 (DOK3) inhibits DAP12, resulting in reduced osteoclast formation (Cai et al. 2017). Patients with polycystic lipomembranous osteodysplasia with sclerosing leukoencephalopathy (PLOSL) exhibit functional defects in DAP12 or TREM-2 (Paloneva et al. 2000). Under stimulation of RANKL and M-CSF, peripheral blood mononuclear cells isolated from PLOSL patients fail to differentiate into osteoclasts properly, showing inefficient and delayed osteoclast differentiation (Cella et al. 2003; Paloneva et al. 2003). Humphrey et al. found that mice with DAP12 deficiency exhibit a mild increase in bone mass. The skeletal tissue lacks osteoclasts, and the formation of multinucleated osteoclasts is impaired, resulting in reduced bone resorption (Humphrey et al. 2004; Kaifu et al. 2003; Nataf et al. 2005; Zou et al. 2008, 2010). Nataf et al. observed the upregulation of TREM-2 in the synovium of active RA, followed by downregulation in the synovium of inactive RA. This suggests that TREM-2 plays a role in RA-induced synovial inflammation (Crotti et al. 2012). Sundaram et al. reported that mononuclear cells from patients with Paget’s disease of bone (PDB) exhibit high levels of SIRPβ1 mRNA expression. Additionally, the measles virus nucleocapsid protein (MVNP) induces an increase in osteoclasts by enhancing the interaction between SIRPβ1 and DAP12.

### Btk/Tec and PLCγ2

The phosphorylated ITAM recruits Syk, which in turn recruits the Tec kinase Btk/Tec and PLCγ. This facilitates the assembly of the osteoclast signaling complex through the activation of adaptor proteins BLNK and SLP76. PLCγ2 is then activated to hydrolyze phosphatidylinositol-4,5-diphosphate, producing inositol-1,4,5-triphosphate (InsP3) and diglycerides (DAG). InsP3 triggers the release of Ca^2+^ from the endoplasmic reticulum, causing an increase in intracellular Ca^2+^ levels. Elevated Ca^2+^ levels activate calmodulin, which leads to the phosphorylation of CaN and the subsequent activation of Ca^2+^/calmodulin-dependent protein kinase (CaMKs9). Ultimately, CaN dephosphorylates serine residues on NFATc1, facilitating its nuclear translocation (Takayanagi 2007). Wang et al. observed significant changes in the expression of Btk, PLCγ2, and NFATc1 during the progression of bone destruction. Silencing Btk resulted in a notable inhibition of osteoclast differentiation, as well as a reduction in the expression of PLCγ2 and NFATc1. These findings suggest that Btk and PLCγ2 are key factors involved in the onset of bone destruction (Wang et al. 2019). Zhuang et al. discovered that the Sema6A-plexin-A2 axis mediates NFATc1 activation via PLCγ signaling, thereby promoting osteoclast formation (Zhuang et al. 2019).

Trang et al. found that citropten pretreatment inhibited the PLCγ/Ca^2+^ signaling pathway and found that it interacted with the active site of proteins in the Ca^2+^ signaling pathway with negative binding affinity, suggesting that citropten is a potential candidate for the treatment of bone destruction (Trang et al. 2023). Park et al. found that β-boswellic acid (βBA), a natural compound found in *Boswellia serrata*, significantly inhibited the phosphorylation of Btk and PLCγ2. It also reduced the expression of NFATc1 at both mRNA and protein levels, resulting in the attenuation of osteoclast differentiation and formation (Park et al. 2021). Jeong et al. discovered that betulinic acid (BA), a natural plant-derived pentacyclic triterpenoid compound, effectively inhibited osteoclastogenesis by suppressing the phosphorylation of the PLCγ2-Ca^2+^ signaling pathway (Jeong et al. 2020a). Zeng et al. found that treatment with artesunate significantly inhibited LPS-induced Ca^2+^ influx and downregulated the expression of PP2B-Aα (calcineurin) and phosphorylated PLCγ1 in cells (Zeng et al. 2020). Ye et al. found that berberine hydrochloride reduced the expression of Ca^2+^-regulated phosphatase and PLCγ, thereby suppressing the TRAF6-Ca^2+^-calcineurin-NFATc1 signaling pathway. This inhibition led to the suppression of osteoclastogenesis and bone destruction (Ye et al. 2017). Baek et al. found that methyl gallate significantly inhibited osteoclast formation by blocking Akt and Btk-PLCγ2-Ca^2+^ signaling (Baek et al. 2017) (Table 2).

**Table 2:** Diverse agents that regulate Ca^2+^-related signaling pathway in osteolclastogenesis

Agent	Mechanism	Cells	Animals	Administered dose		References
				In vitro	In vivo	
Citropten	Inhibited MAPK and PLCγ/Ca^2+^ signaling pathways	RAW264.7 cell	N/A	5–40 μM	N/A	Trang et al. (2023)
βBA	Inhibited the phosphorylation of Btk and PLCγ2	BMMs (ICR mice)	N/A	5–30 μM	N/A	Park et al. (2021)
BA	Attenuated the phosphorylation of PLCγ2-Ca^2+^ signaling pathway	BMMs (ICR mice)	ICR mice	1–10 μM	10 mg/kg	Jeong et al. (2020a)
Artesunate	Inhibited Ca^2+^ influx and decreased the expression of PP2B-Aα (calcineurin) and pPLCγ1	RAW264.7 cell	ICR mice	3.125–12.5 μM	10 mg/kg	Zeng et al. (2020)
Berberine hydrochloride	Reduced the expression of Ca^2+^-regulated phosphatase and PLCγ	RAW264.7 cell	N/A	5–20 μM	N/A	Ye et al. (2017)
Methyl gallate	Blocked the Akt and Btk-PLCγ2-Ca^2+^ signaling	BMMs (ICR mice)	ICR mice	1–10 μM	10 mg/kg	Baek et al. (2017)

In summary, the interaction between RANK and its co-stimulatory immune receptors plays a crucial role in mediating the Ca^2+^ signaling pathway. This pathway involves a series of reactions including recruitment and hydrolysis, which ultimately leads to the stable amplification of NFATc1 and promotes the occurrence of bone destruction.

## Bcl-6, IRF8, MafB, and Lhx2 negatively regulate the expression of NFATc1

**Fig. 4:**
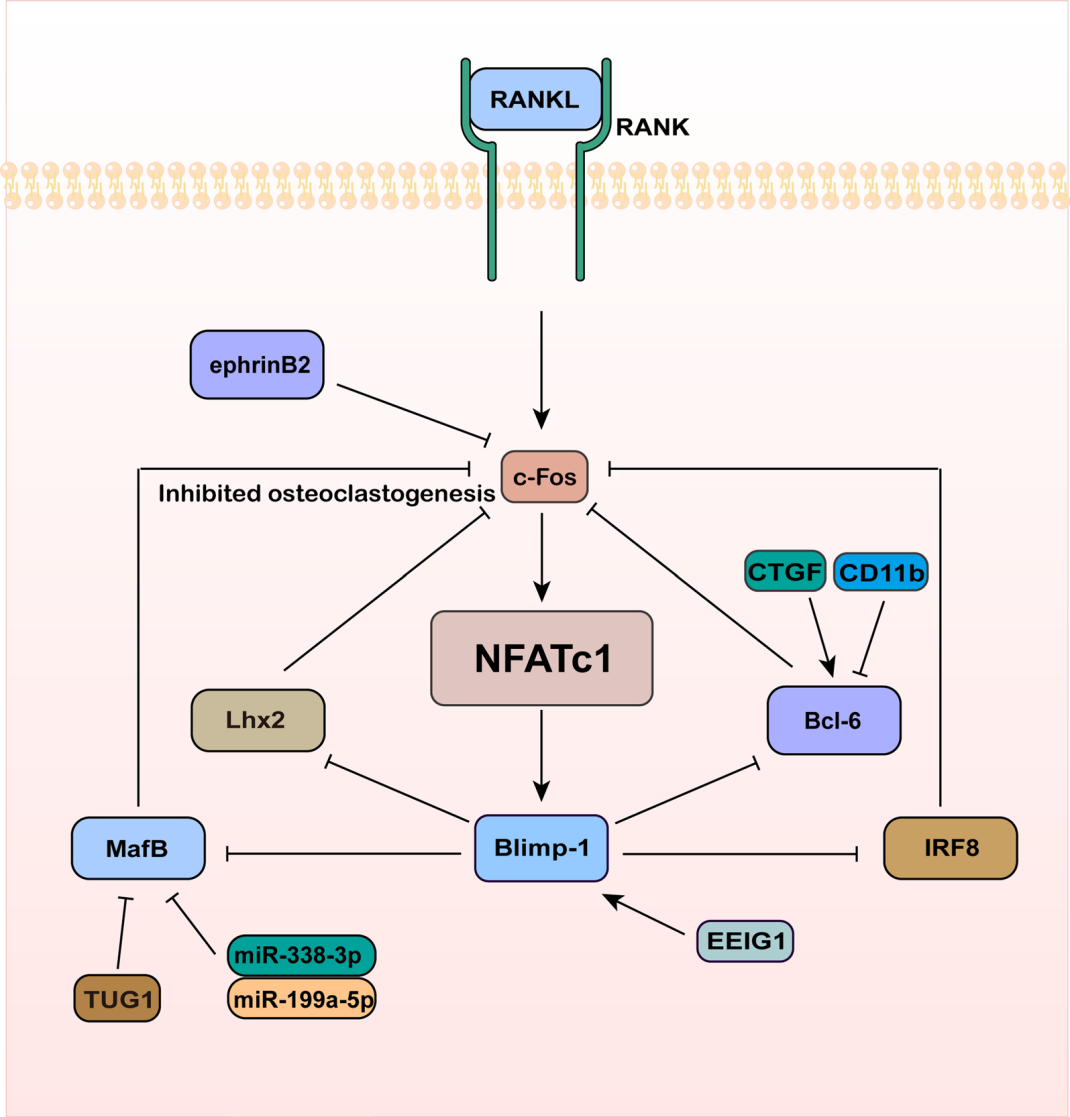
Bcl-6, IRF8, MafB, and Lhx2 negatively regulate the expression of NFATc1. In osteoclast differentiation, Bcl-6, IRF8, MafB, Lhx2, and ephrinB2 negatively regulate NFATc1 by inhibiting c-Fos expression. Blimp-1 can suppress the expression of Bcl-6, IRF8, MafB, and Lhx2. CTGF activate but CD11b inhibit Bcl-6. EEIG1 combines with Blimp-1 to downregulate IRF8. TUG1, miR-338-3p, and miR-199a-5p inhibit Maf8

When NFATc1 is continuously activated by Ca^2+^ signaling, it inhibits negative regulators of NFATc1. B lymphocyte-induced maturation protein-1 (Blimp-1) is one of the negative regulatory factors. It downregulates the expression of NFATc1 negative regulators such as Bcl-6, IRF8, MafB, and Lhx2 (Kim et al. 2007; Nishikawa et al. 2010; Zhao et al. 2009). NFAT proteins consist of an N-terminal transactivation domain, a regulatory domain, a DNA-binding domain, and a C-terminal transactivation domain (Zhang et al. 2016). Lhx2 can interact with c-Fos and weaken its DNA-binding ability, thereby inhibiting the transactivation of NFATc1 (Kim et al. 2014). Bcl-6 suppresses the expression of genes involved in osteoclast function, such as dendritic cell-specific transmembrane protein (DC-STAMP), NFATc1, and CTSK, leading to the inhibition of osteoclast differentiation. This is substantiated by the findings that mice lacking Bcl-6 exhibit heightened osteoclast activity and reduced bone mass (Miyamoto 2011). When there is an appropriate level of osteoclasts present, Bcl-6 exhibits high expression in Blimp-1 knockout mice, leading to the inhibition of osteoclast differentiation. This suggests that Blimp-1 suppresses the expression of Bcl-6, and Bcl-6, in turn, inhibits the expression of osteoclast genes, thus forming a negative regulatory loop involving NFATc1, Blimp-1, and Bcl-6. Choi et al. found that treating osteoclasts with connective tissue growth factor (CTGF) leads to downregulation of Bcl-6 mRNA and protein expression. This finding demonstrates that CTGF promotes osteoclast differentiation by reducing Bcl-6 levels and increasing DC-STAMP expression (Choi et al. 2020). Park et al. found that CD11b inhibited NFATc1 activation by downregulating RANK expression and inducing Bcl-6 recruitment to the NFATC6 gene, thus acting as a negative regulator in the early stages of osteoclast differentiation (Park-Min et al. 2013). Jeong et al. found that early estrogen-induced gene 1 (EEIG1) as a negative regulator of osteoclast differentiation. EEIG1 forms a complex with Blimp-1 and negatively regulates the expression of the osteoclast-resistant gene IRF8, promoting osteoclast differentiation (Jeong et al. 2020b). Saito et al. observed that specific knockout of IRF8 enhanced osteoclast differentiation and bone resorption when stimulated with M-CSF and RANKL (Saito et al. 2017). Han et al. generated a mutant zebrafish lacking the MafB homolog Mafbb using CRISPR/Cas9 and found that Mafbb-deficient zebrafish exhibited enhanced osteoclast differentiation and formation (Han et al. 2021). Du et al. discovered that the long non-coding RNA TUG1 was overexpressed during osteoclast differentiation and positively regulated osteoclast formation by targeting MafB (Du et al. 2020). Sun et al. discovered that miR-338-3p acts as a regulatory factor for MafB by targeting and suppressing its gene expression. RNA silencing of MafB can block the pro-differentiation effect of miR-338-3p on osteoclasts (Sun et al. 2019). Guo et al. found that miR-199a-5p activated NFATc1 by suppressing MafB gene expression, resulting in a positive regulation of osteoclast differentiation (Guo et al. 2019). Additionally, NFATc1 induces the transcription of hepatic ligand protein B2 (EphrinB2), which inhibits osteoclast differentiation by downregulating c-Fos in osteoclast precursor cells. Therefore, ephrinB2 is considered a potential negative regulator of NFATc1 (Matsuo and Otaki 2012) (Fig. 4).

Fang et al. demonstrated that Unkeito (UKT), a Kampo medicine, inhibited osteoclastogenesis and increased mononuclear osteoclast apoptosis by reducing Blimp-1 expression and elevating Bcl-6 expression (Fang et al. 2022). Cao et al. found that knockdown of Blimp-1 in bone marrow-derived macrophages (BMMs) using siRNA significantly enhanced Bcl-6 expression and decreased NFATc1 expression. Their findings suggest that agrimophol (AGR), a phenolic compound derived from *Agrimonia pilosa Ledeb*, mediated NFATc1 expression and its regulation of target genes through the Blimp-1-Bcl-6 signaling pathway, thereby inhibiting osteoclast differentiation (Cao et al. 2021). Jin et al. discovered that Gs, one of the oligomers of glucuronomannan, attenuated the degradation of IRF8 and downregulated NFATc1 expression, resulting in the inhibition of osteoclast differentiation (Jin et al. 2023a). Zhang et al. observed that treatment of BMMs with water-based purple tea extract (PTE) reduced Blimp-1 expression and increased IRF8 levels, which subsequently inhibited NFATc1 expression and osteoclast differentiation (Zhang et al. 2022). Gao et al. found elevated levels of long non-coding RNA maternally expressed gene 3 (MEG3) and decreased levels of IRF8 in an osteolytic mouse model. They also demonstrated that IL-10 could inhibit MEG3 expression and increase IRF8 levels by promoting MEG3 methylation, indicating that IL-10 acted as an inhibitor of osteoclast differentiation (Gao et al. 2022) (Table 3).

**Table 3:** Diverse agents that regulate the negative regulators of NFATc1 in osteolclastogenesis

Agent	Mechanism	Cells	Animals	Administered dose		References
				In vitro	In vivo	
UKT	Decreased Blimp-1 expression and elevated Bcl-6 expression	RAW264.7 cell	CD-1 mice	50–500 μg/mL	N/A	Fang et al. (2022)
AGR	Inhibited Blimp-1 expression and activated Bcl-6 expression	BMMs (C57BL/6 mice)	C57BL/6 mice	1.25–10 μM	10 mg/kg	Cao et al. (2021)
Gs	Attenuated the degradation of IRF8 and downregulated NFATc1 expression	RAW264.7 cell	N/A	10–50 μg/mL	N/A	Jin et al. (2023a)
PTE	Decreased Blimp-1 expression and increased IRF8 levels	BMMs (C57BL/6 mice)	C57BL/6 mice	6.125–50 μg/mL	30 mg/kg	Zhang et al. (2022)
IL-10	Inhibited MEG3 expression and increased IRF8 levels	RAW264.7 cell BMMs (C57BL/6 mice)	C57BL/6 mice	100 ng/mL	N/A	Gao et al. (2022)

Collectively, the negative regulators of NFATc1 play a crucial role in osteoclast differentiation and can mitigate bone destruction by inhibiting NFATc1 expression. Targeting these negative regulators offers a potential approach to alleviate bone destruction. Additionally, restoring osteoclast formation by inhibiting the negative regulators of NFATc1 represents a novel strategy for treating certain diseases characterized by impaired osteoclast differentiation, such as osteonecrosis (Du et al. 2023).

## Epigenetic regulation of NFATc1

In addition to the three major regulatory mechanisms, epigenetic modification is involved in the regulation of NFATc1 in osteoclasts. Epigenetics refers to the study of heritable changes in the function of genetic elements without alterations in the DNA sequence (Bird 2007). There are three classes of epigenetic markers: DNA methylation, histone modification, and noncoding RNAs. These markers play a crucial role in determining cell fate (Yasui et al. 2011a).

DNA methylation is the most well-known method of epigenetic modification. In general, hypermethylation inhibits gene expression, while hypomethylation promotes it (Jaenisch and Bird 2003; Yasui et al. 2011a). Yasui et al. demonstrated through ChIP-seq that histone H3 lysine 4 trimethylation (H3K4me3) was expressed in the NFATc1 gene in osteoclast precursor cells, but its expression was significantly reduced in mature osteoclasts. During osteoclast differentiation, the expression and recruitment of H3K27 demethylase macrophage structural domain protein 3 (Jmjd3) is induced around the transcription start site of NFATc1. Inhibition of Jmjd3 using short hairpin RNA down-regulates osteoclast differentiation by preventing demethylation of H3K27me3 at the transcription start site of NFATc1 (Yasui et al. 2011b). Integrin subunit β 3 (ITGB3) serves as an osteoclast marker. Yu et al. detected H3K9 monomethylation (H3K9me1) and H3K9 dimethylation (H3K9me2) modifications, as well as lysine specific demethylase 1 (LSD1) protein enrichment in the ITGB3 promoter by ChIP-seq. The study by Yu et al. suggests that LSD1 promotes ITGB3 expression by decreasing the level of H3K9 in the ITGB3 promoter. Additionally, LSD1 enhances ITGB3 expression by decreasing the levels of H3K9me1 and H3K9me2 in the ITGB3 promoter, which in turn promotes the expression of NFATc1 and osteoclast formation (Yu et al. 2023a). Stegen et al. found that the activity of a histone demethylase requires α-ketoglutarate derived from the serine synthesis pathway (SSP). This enzyme induces NFATc1 expression and subsequent osteoclast maturation by removing the inhibitory histone methylation mark from the NFATc1 gene locus (Stegen et al. 2024). Yang et al. demonstrated through meRIP-Seq that exosome-released methyltransferase-like 14 (METTL14) enhances the m6A methylation level of NFATc1, which in turn suppresses the transcription of NFATc1, thereby reducing osteoclastogenesis (Yang et al. 2023). Wang et al. showed that early growth response protein 1 (EGR1) promotes METTL3 transcription and increases the level of m6A-modified chitinase-3-like protein 1 (CHI3L1), thereby stressing osteoclast differentiation (Wang et al. 2023a). These results suggest that regulating NFATc1 expression by methylation or demethylation mechanisms, which in turn affects osteoclast differentiation, is a feasible strategy.

Post-translational modifications, such as acetylation and ubiquitination, play a crucial role in gene regulation (Bae and Lee 2006). Shalev et al. discovered that protein tyrosine phosphatase receptor type J (PTPRJ) promotes osteoclast differentiation by decreasing ubiquitination and degradation of the key osteoclast transcription factor NFATc1 through dephosphorylation of M-CSF receptor (M-CSFR) and Cbl. The deletion of PTPRJ increases the ubiquitination of NFATc1 and decreases its expression, thereby inhibiting femoral cell maturation (Shalev et al. 2021). Narahara et al. found that Kelch repeat and BTB domain-containing protein 11 (KBTBD11) interacts with the E3 ubiquitin ligase Cullin3, promoting the ubiquitination and degradation of NFATc1 by the proteasome. This finding confirms that KBTBD11 is a negative regulator of osteoclast differentiation by controlling Cullin3-mediated ubiquitination of NFATc1 (Narahara et al. 2019). Kim et al. found that M-CSF inhibites osteoclastogenesis by inducing NFATc1 ubiquitination and degradation via (calcineurin B like) Cbl proteins in a Src kinase-dependent manner at late stages of osteoclast differentiation (Kim et al. 2010). NFATc1 acetylation is mediated by P300/CBP-associated factor (PCAF)’s histone acetyltransferase (HAT) activity through its interaction with NFATc1. Histone deacetylase 5 (HDAC5) significantly inhibits NFATc1 acetylation in the HDAC proteome. Additionally, HDAC5 decreases the stability and transactivation of NFATc1, thereby inhibiting osteoclast differentiation. RANKL can promote NFATc1 accumulation by inducing its acetylation (Kim et al. 2011). Therefore, the cytokines M-CSF and RANKL play a crucial role in osteoclast differentiation by respectively inducing ubiquitination and acetylation of NFATc1, which regulates its stability and activity (Kim and Kim 2014).

Long-stranded non-coding RNAs known as lncRNAs, are enriched with more than 200 nucleotides and act as messengers to regulate biological activities such as cell proliferation, migration, and differentiation through corresponding signaling pathways (Zhang et al. 2023a). MiRNAs, on the other hand, are miniaturized non-coding RNAs with lengths ranging from 22 to 28 nucleotides. They typically bind to the 3′UTR region of target genes and regulate their expression levels. MicroRNAs play various roles in cell proliferation, differentiation, and apoptosis (Ambros 2004; Bartel 2004). Zhang et al. found that LncRNA metastasis-associated lung adenocarcinoma transcript 1 (MALAT1) promotes osteoclastogenesis by elevating NFATc1 expression through competing for miRNA-124 binding, acting as an endogenous sponge (Zhang et al. 2023a). Zhang et al. found that LncRNA KCNQ1 opposite strand/antisense transcript 1 (Kcnq1ot1) reduces NFATc1 expression and inhibits osteoclast differentiation (Zhang et al. 2021b). Takigawa et al. found that miR-222-3p could reduce the expression of NFATc1 by blocking the activity of c-Src, thus inhibiting osteoclastogenesis (Takigawa et al. 2016). Although the current study has shown that several non-coding RNAs can be involved in regulating osteoclast formation, further studies are still needed to find one that can directly regulate NFATc1 expression in osteoclasts.

## Drug likeness analysis

**Fig. 5:**
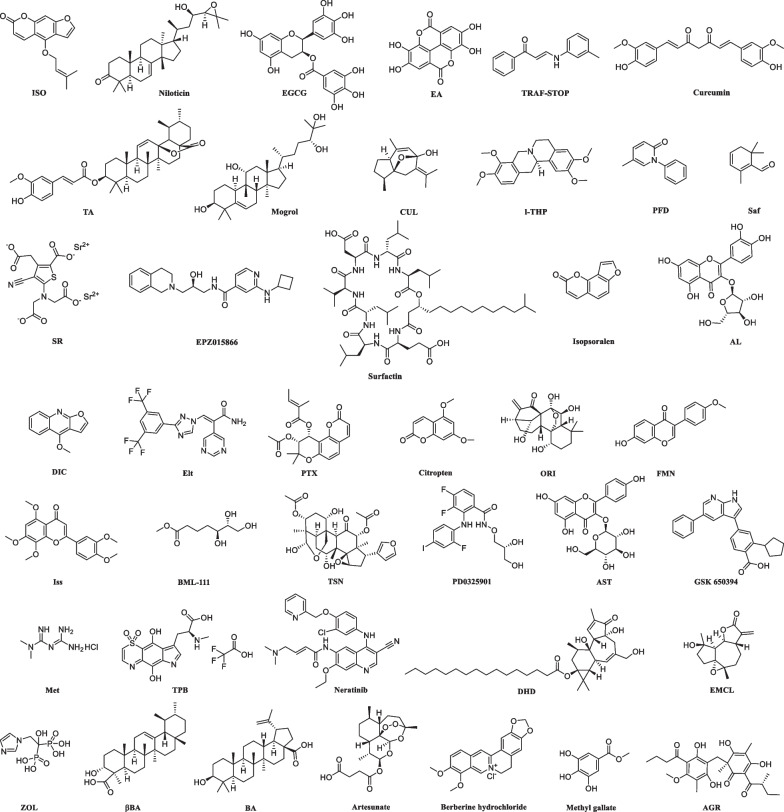
Structures of the 41 agents that target NFATc1

**Fig. 6:**
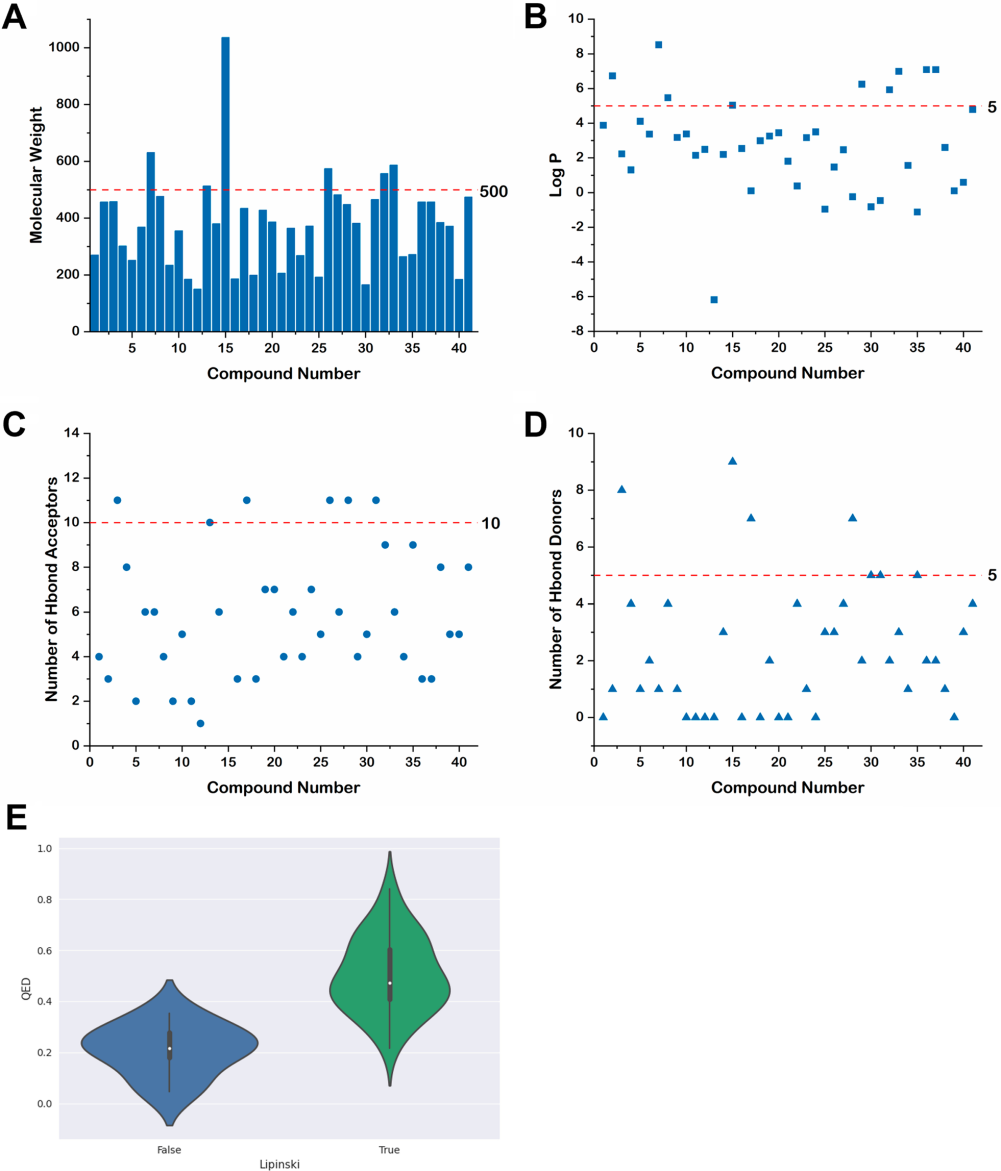
Drug likeness analysis of the agents that targets NFATc1. **A** Molecular weight. **B** Octanol–water partition coefficient. **C** Number of hydrogen bond acceptors. **D** Number of hydrogen bond donors. **E** QED value

For the 52 agents listed above, we found the structures of the compounds corresponding to 41 of them through literature search (Fig. 5) and analyzed them for drug likeness by using Python’s rdkit package (Goodwin et al. 2017; Lazic et al. 2022; Liu et al. 2023a; Liu et al. 2022b). Commonly, the drug properties of chemical molecules can be initially determined according to Lipinski’s rule V. Lipinski’s rule V is defined as a molecular weight not exceeding 500 Dalton, octanol–water partition coefficient [log P] not exceeding 5, hydrogen bond acceptor not exceeding 10, and hydrogen bond donor not exceeding 5 (Lipinski et al. 2001). The results of our analysis showed that of the 41 compounds, 35 compounds had molecular weights not exceeding 500 (Fig. 6A). 32 compounds had log P values not exceeding 5 (Fig. 6B). 35 compounds had no more than 10 hydrogen bond acceptors (Fig. 6C). 37 compounds had no more than 5 hydrogen bond donors (Fig. 6D). In summary, 33 compounds (63%) met all criteria for Lipinski’s rule V, and only 8 compounds (37%) failed the Lipinski’s rule V (Tab. 4). Among these compounds, l-THP showed highest quantitative estimate of drug-likeness (QED) value as 0.84, and Surfactin showed lowest QED value as 0.05 (Fig. 6E). Although these agents are effective in inhibiting RA bone destruction by targeting NFATc1, further studies are needed to determine which ones have the potential to be developed into drugs.

**Table 4:** Drug likeness analysis of the agents that targets NFATc1

No.	Compounds	Molecular formula	QED	Lipinski	Molecular weight	Log P	HBA	HBD	References
1	ISO	C_16_H_14_O_4_	0.53	True	270.28	3.88	4	0	Fan et al. (2023); Li et al. (2023)
2	Niloticin	C_30_H_48_O_3_	0.38	True	456.71	6.73	3	1	Xu et al. (2022)
3	EGCG	C_22_H_18_O_11_	0.21	False	458.38	2.23	11	8	Xu et al. (2021)
4	EA	C_14_H_6_O_8_	0.22	True	302.19	1.31	8	4	Xu et al. (2020)
5	TRAF-STOP	C_17_H_17_NO	0.65	True	251.33	4.11	2	1	Huang et al. (2023)
6	Curcumin	C_21_H_20_O_6_	0.55	True	368.39	3.37	6	2	Ke et al. (2023); von Metzler et al. (2009)
7	TA	C_40_H_54_O_6_	0.20	False	630.87	8.52	6	1	Liu et al. (2022a)
8	Mogrol	C_30_H_52_O_4_	0.40	True	476.74	5.47	4	4	Chen et al. (2022b)
9	CUL	C_15_H_22_O_2_	0.65	True	234.34	3.18	2	1	Wang et al. (2021)
10	l-THP	C_21_H_25_NO_4_	0.84	True	355.43	3.38	5	0	Zhi et al. (2020)
11	PFD	C_12_H_11_NO	0.67	True	185.23	2.15	2	0	Zhang et al. (2023b)
12	Saf	C_10_H_14_O	0.52	True	150.22	2.49	1	0	Sheng et al. (2023)
13	SR	C_12_H_6_N_2_O_8_SSr_2_	0.30	True	513.49	− 6.18	10	0	Wu et al. (2023)
14	EPZ015866	C_22_H_28_N_4_O_2_	0.69	True	380.49	2.20	6	3	Ding et al. (2023)
15	Surfactin	C_53_H_93_N_7_O_13_	0.05	False	1036.36	5.04	20	9	Kuang et al. (2023)
16	Isopsoralen	C_11_H_6_O_3_	0.51	True	186.17	2.54	3	0	Zhan et al. (2023); Zhu et al. (2023)
17	AL	C_20_H_18_O_11_	0.28	False	434.35	0.10	11	7	Zhuang et al. (2023)
18	DIC	C_12_H_9_NO_2_	0.60	True	199.21	2.99	3	0	Wong et al. (2022)
19	Elt	C_17_H_10_F_6_N_6_O	0.51	True	428.30	3.26	7	2	Chen et al. (2022a)
20	PTX	C_21_H_22_O_7_	0.45	True	386.40	3.45	7	0	Sun et al. (2023)
21	Citropten	C_11_H_10_O_4_	0.70	True	206.20	1.81	4	0	Trang et al. (2023)
22	ORI	C_20_H_28_O_6_	0.46	True	364.44	0.38	6	4	Jin et al. (2023b)
23	FMN	C_16_H_12_O_4_	0.78	True	268.27	3.17	4	1	Ni et al. (2023); Yu et al. (2023b)
24	Iss	C_20_H_20_O_7_	0.66	True	372.37	3.50	7	0	Qin et al. (2023)
25	BML-111	C_8_H_16_O_5_	0.47	True	192.21	− 0.96	5	3	Wang et al. (2023b)
26	TSN	C_30_H_38_O_11_	0.35	False	574.62	1.47	11	3	Tan et al. (2023)
27	PD0325901	C_16_H_14_F_3_IN_2_O_4_	0.36	True	482.20	2.47	6	4	Jiang et al. (2023)
28	AST	C_21_H_20_O_11_	0.28	False	448.38	− 0.24	11	7	Jia et al. (2019); Xing et al. (2022)
29	GSK 650394	C_25_H_22_N_2_O_2_	0.44	True	382.46	6.25	4	2	Jin et al. (2022)
30	Met	C_4_H_12_ClN_5_	0.32	True	165.63	− 0.82	5	5	Chen et al. (2022c)
31	TPB	C_16_H_14_F_3_N_3_O_8_S	0.37	True	465.36	− 0.46	11	5	Wang et al. (2022d)
32	Neratinib	C_30_H_29_ClN_6_O_3_	0.22	False	557.05	5.93	9	2	Qiu et al. (2022)
33	DHD	C_36_H_58_O_6_	0.10	False	586.85	6.99	6	3	He et al. (2022b)
34	EMCL	C_15_H_20_O_4_	0.41	True	264.32	1.57	4	1	Long et al. (2022)
35	ZOL	C_5_H_10_N_2_O_7_P_2_	0.43	True	272.09	− 1.12	9	5	Huang et al. (2022)
36	βBA	C_30_H_48_O_3_	0.41	True	456.71	7.09	3	2	Park et al. (2021)
37	BA	C_30_H_48_O_3_	0.44	True	456.71	7.09	3	2	Jeong et al. (2020a)
38	Artesunate	C_19_H_28_O_8_	0.58	True	384.43	2.60	8	1	Zeng et al. (2020)
39	Berberine hydrochloride	C_20_H_18_ClNO_4_	0.60	True	371.82	0.10	5	0	Ye et al. (2017)
40	Methyl gallate	C_8_H_8_O_5_	0.44	True	184.15	0.59	5	3	Baek et al. (2017)
41	AGR	C_26_H_34_O_8_	0.30	True	474.55	4.79	8	4	Cao et al. (2021)

## Conclusions

The inflammatory response in RA patients often leads to abnormal proliferation of osteoclasts, which disrupts the balance between osteoclasts and osteoblasts, thus causing bone destruction and resulting in disability. NFATc1 is the key transcriptional regulator in osteoclastogenesis and plays a crucial role in osteoclast differentiation, formation, and fusion. Additionally, NFATc1 can directly activate osteoclasts and promote their differentiation. Therefore, it is essential to restore the balance of the bone environment by regulating NFATc1 to inhibit osteoclast differentiation. In this review, we summarize the regulatory mechanisms and recent advances of NFATc1 in bone destruction, and list its potential agents, aiming to provide some valuable insights for future studies in the field of RA. By comparing the results of these agents in cells of animal and human or in animal models, we found that although some of the agents showed good therapeutic effects on RA bone destruction in animal models and met the preliminary drug properties, further investigation is needed to determine their potential for development into drugs and application in the treatment of RA patients.

## Data Availability

All of the original data are available with the corresponding author and can be provided upon reasonable request.
